# Aggravated stuttering following subthalamic deep brain stimulation in Parkinson's disease - two cases

**DOI:** 10.1186/1471-2377-11-44

**Published:** 2011-04-08

**Authors:** Mathias Toft, Espen Dietrichs

**Affiliations:** 1 Department of Neurology, Oslo University Hospital, Oslo, Norway; 2 Faculty of Medicine, University of Oslo, Oslo, Norway

**Keywords:** Parkinson's disease, deep brain stimulation, subthalamic nucleus, stuttering, verbal fluency

## Abstract

Stuttering is a speech disorder with disruption of verbal fluency which is occasionally present in patients with Parkinson's disease (PD). Long-term medical management of PD is frequently complicated by fluctuating motor functions and dyskinesias. High-frequency deep brain stimulation (DBS) of the subthalamic nucleus (STN) is an effective treatment of motor fluctuations and is the most common surgical procedure in PD. Here we report the re-occurrence and aggravation of stuttering following STN-DBS in two male patients treated for advanced PD. In both patients the speech fluency improved considerably when the neurostimulator was turned off, indicating that stuttering aggravation was related to neurostimulation of the STN itself, its afferent or efferent projections and/or to structures localized in the immediate proximity. This report supports previous studies demonstrating that lesions of the basal ganglia-thalamocortical motor circuit, including the STN, is involved in the development of stuttering. In advanced PD STN-DBS is generally an effective and safe treatment. However, patients with PD and stuttering should be informed about the risk of aggravated symptoms following surgical therapy.

## Background

Stuttering is a speech disorder with disruption of verbal fluency characterized by involuntary repetitions or prolongations of sounds or syllables. The pathogenesis of stuttering is not well understood. The most common form of stuttering is developmental stuttering, which evolves in childhood. Neurogenic or acquired stuttering occurs after a definable brain damage, e.g. stroke, intracerebral hemorrhage, or head trauma [1]. Acquired stuttering has been observed after lesions in a variety of brain areas, including lesions in the striatum or thalamus. The occurrence of basal ganglia disorders, such as Parkinson's disease (PD), occasionally leads to a re-emergence of recovered developmental stuttering [2].

PD is a movement disorder defined by the presence of the cardinal motor features bradykinesia, rigidity and tremor. Long-term medical management of PD is frequently complicated by treatment-associated motor complications in the form of fluctuating motor function and dyskinesias. High-frequency deep brain stimulation (DBS) of the subthalamic nucleus (STN) is an effective treatment in advanced PD, improving all cardinal motor symptoms and treatment-related motor complications [3].

DBS of different parts of the basal ganglia and thalamus have become widely applied surgical procedures. Observations on surgical and target related side effects have provided novel insights into the function of these parts of the brain. STN-DBS is currently the most common surgical procedure for patients with PD suffering from intolerable motor complications despite optimal medical treatment. Here we report the re-occurrence and aggravation of stuttering following STN-DBS in two male patients treated for advanced PD. This supports previous studies indicating a role for the STN in the development of stuttering.

## Case presentations

### Patient 1

This 65-year-old right-handed man was diagnosed with PD at the age of 40 years. The disease had initially started with resting tremor on the right side, with subsequent development of rigidity and bradykinesia. In parallel with the parkinsonian symptoms he had developed stuttering. He had no history of developmental stuttering. However, he reported a history of an abnormally rapid speech since his childhood. After more than 20 years of excellent response to dopaminergic treatments he developed more marked motor fluctuations, with tremor and freezing of gait in periods of the day with poor effect of medication (off-periods), and troublesome dyskinesias during periods with good medication effect (on-periods). When preoperative evaluation for DBS was performed he was taking 2 mg of cabergoline and a total of 450 mg levodopa in combination with 1400 mg of entacapone. After an overnight withdrawal of dopaminergic treatment he had a Unified Parkinson's Disease Rating Scale (UPDRS) motor score of 33 in the off-medication state. In the on-medication state this score was 12. At the age of 63 years surgery was performed using our standard procedure with bilateral implantation of STN electrodes targeted in accordance with preoperative MRI and intraoperative microrecordings typical for the STN region.

After surgery the patient had marked improvement of all parkinsonian cardinal features. Freezing of gait and dyskinesias disappeared and there was a clear reduction in motor fluctuations. However, the patient recognized a marked worsening of speech fluency under effective neurostimulation. One month after surgery the UPDRS motor score in the on-stimulation off-medication state was 12. The medication was reduced to 200 mg of levodopa in combination with 800 mg entacapone. Stimulation parameters were monopolar stimulation with contacts 1 and 5 as cathodes with bilateral amplitudes of 3.0 V, 60 μs pulse width and a frequency of 130 Hz. With these settings he had severe stuttering. The main problem consisted in difficulties initiating speech. Singing clearly reduced the stuttering. He had no hypophonia or other speech abnormalities. No other side effects occurred. The influence of stimulation settings on stuttering was assessed both during spontaneous speech and during reading, and the examination was videotaped with the patient and one of the authors (ED) blinded to the stimulation parameters. After switching the neurostimulator off, stuttering severity decreased quickly over seconds. Programming sessions were undertaken in order to optimize the stimulation parameters, but aggravation of stuttering was clearly related to stimulation-induced motor improvements.

At the last examination two years after the procedure the speech dysfluency has showed some improvement. Nevertheless, the patient occasionally switches the device off during some activities as phone calls and public talks. Stimulation parameters remained unchanged with amplitudes of 3.0 V, 60 μs pulse width and a frequency of 130 Hz. The medication was 300 mg of levodopa in combination with 800 mg entacapone.

### Patient 2

A 60-year-old right-handed man was diagnosed with PD at the age of 38 years. Six years after receiving the diagnoses he was treated with unilateral thalamotomy in the left brain hemisphere. The disease gradually progressed and he eventually developed marked rigidity, moderate bradykinesia, but the tremor remained mild. He was evaluated for DBS because of marked motor fluctuations with troublesome dyskinesias. He was then taking a total of 1.08 mg pramipexol, 900 mg of levodopa and 900 mg of entacapone daily. UPDRS motor scores were 35 in the off-medication state and 8 in the on-medication state. At the age of 53 years DBS electrodes were implanted in the STN bilaterally.

Subthalamic stimulation had marked effects on all cardinal symptoms, and in the first months after surgery the patient experienced no motor fluctuations or dyskinesias. The UPDRS motor score in the on-stimulation off-medication state one month after surgery was 14. He had stopped taking pramipexol and entacapone, but the daily levodopa intake had remained at 900 mg per day. The patient complained about marked increase in stuttering, a symptom which he had experienced in his childhood and that re-emerged after he developed PD. In addition, the patient also experienced dysarthria and hypophonia, although these symptoms did not worsen after the implantation. The relationship between stuttering and neurostimulation was examined with the rater blinded to stimulation parameters. Stuttering was clearly related to neurostimulation and improvement of parkinsonian symptoms. The best motor improvements were achieved with monopolar stimulation with contacts 2 and 6 as cathodes, bilateral amplitudes of 2.6 V, pulse width 60 μs, and a frequency of 130 Hz. The patient received intensive speech therapy and stuttering gradually improved over the first two years after surgery. At the latest examination seven years after implantation the UPDRS motor scores were 21 in the on-stimulation off-medication and 11 in the on-stimulation on-medication states. He was then taking a total of 1250 mg of levodopa daily.

## Conclusions

We describe two cases of aggravated stuttering after STN-DBS in our series of 144 PD patients operated between January 2001 and December 2007. One of the patients had a history of developmental stuttering, whereas the other patient first developed stuttering years after receiving a diagnosis of PD. The main problem for both patients was difficulty in speech initiation, with blocks and repetitions at word onset. Stuttering improved somewhat in the first postoperative months, but remained a problem in daily life for patient 1. The effect of neurostimulation on stuttering was clearly associated with its effects on motor functions in these two patients, and both patients had marked improvements of motor fluctuations and dyskinesias. In addition, in both patients the speech fluency improved considerably when the neurostimulator was turned off. This indicates that the electrodes were correctly placed, and that stuttering aggravation was related to neurostimulation of the STN itself, its afferent or efferent projections, and/or to structures localized in the immediate proximity.

Stuttering is a speech dysfluency. Verbal fluency tests are often used to assess executive cognitive function, and a decline in verbal fluency is consistently reported following DBS for PD. Virtually all surveys assessing phonological and/or semantic controlled word association tasks after STN-DBS report a significant and long-standing worsening of performance with respect to the pre-operative level [4]. Similar findings have also been obtained after pallidal and thalamic surgery [5]. Impaired verbal fluency after DBS has been interpreted as impairment executive functioning [6]. However, the identified aggravation of stuttering indicates that the decline in verbal fluency measured after subthalamic stimulation is, at least partly, not related to executive dysfunction, but a motor dysfunction of speech.

The STN is one of the main regulators of motor function related to the basal ganglia, through its fundamental role within the basal ganglia-thalamocortical motor circuit [7]. This motor circuit is postulated to play a key role in the etiology of stuttering. The main dysfunction is thought to be impairment in the ability of the basal ganglia to produce timing cues for the initiation of the next motor segment of speech [8]. The aggravated stuttering in the two presented patients support that alterations of the mentioned circuit at the level of the STN can influence stuttering, and indicates that the basal ganglia play a central role in speech production. The striking finding that STN-stimulation is able to modulate stuttering severity reversibly underlines the importance of the STN and the basal ganglia network in general for motor activity regulation during normal and abnormal speech.

Our findings are supported by two previous reports of worsening of developmental and acquired stuttering following bilateral STN-DBS. Moretti et al reported speech initiation problems in a PD patient without speech problems prior to DBS surgery [9]. In a report by Burghaus and colleagues a marked worsening of stuttering was found after STN-DBS [10]. In their patient developmental stuttering had re-occurred after the development of PD. As in our patients, the stimulation-induced speech dysfluency slowly improved in the first months after surgery. Notably, unilateral stimulation of the STN in the language-dominant hemisphere has been reported to improve acquired stuttering [11]. It is unknown whether this discrepancy could be explained by different placements of the electrodes in the subthalamic region, differences between unilateral and bilateral stimulation, or different causes of stuttering in the reported patients. The result of unilateral stimulation was unfortunately not tested in our patients. Interestingly, it has recently been reported that DBS of another part of the basal ganglia, the internal globus pallidus, in two patients treated for dystonia also induced stuttering [12]. The sensorimotor GPi is connected to the same parts of the motor circuit as the STN, and in patients with PD stimulation of the GPi has similar effects on motor complications as stimulation in the STN [13]. Thus, alterations of the same network on different places may induce or worsen stuttering.

It seems clear that lesions of the basal ganglia-thalamocortical motor circuit are a frequent cause of neurogenic stuttering [8]. Verbal fluency improves following the administration of haloperidol and other dopamine antagonists, a neurotransmitter in the motor circuit [14]. Dopamine antagonists generally markedly increase bradykinesia and rigidity of the limbs in parkinsonian patients. This indicates that speech and body motor systems might be neuroanatomically distinct. The findings in our two patients support the hypothesis of a STN contribution to speech processes both in developmental and neurogenic stuttering. Interestingly, a striking area of overactivity was recently seen in the midbrain in people with developmental stuttering relative to controls [15].

STN-DBS is generally an effective and safe treatment [3]. However, prior to performing STN-DBS, patients with PD and stuttering should be informed about the risk of aggravated symptoms. Although stimulation-induced worsening of stuttering seemed to improve over time, it could still be a significant problem for the individual patient.

## Consent

Written informed consent was obtained from the patients for publication of these case reports.

## Competing interests

Both authors have received travel grants and honoraria from Medtronic.

## Authors' contributions

MT examined the patients and drafted the manuscript. ED performed blinded video evaluations of patients and helped to draft the manuscript. Both authors read and approved the final manuscript.

## Pre-publication history

The pre-publication history for this paper can be accessed here:

http://www.biomedcentral.com/1471-2377/11/44/prepub
